# Village health volunteers’ social capital related to their performance in Lao People’s Democratic Republic: a cross-sectional study

**DOI:** 10.1186/1472-6963-14-123

**Published:** 2014-03-12

**Authors:** Yu Sato, Tiengkham Pongvongsa, Daisuke Nonaka, Sengchanh Kounnavong, Phetsomphone Nansounthavong, Kazuhiko Moji, Panom Phongmany, Yasuhiko Kamiya, Miho Sato, Jun Kobayashi

**Affiliations:** 1 Graduate School of International Health Development, Nagasaki University, Nagasaki, Japan; 2 Savannakhet Provincial Malaria Station, Savannakhet, Lao People’s Democratic Republic; 3 National Institute of Public Health, Vientiane, Lao People’s Democratic Republic; 4 Savannakhet Provincial Health Department, Savannakhet, Lao People’s Democratic Republic; 5 Sepon District Health Office, Savannakhet, Lao People’s Democratic Republic; 6 Research Institute for Humanity and Nature, Kyoto, Japan; 7 Department of Parasitology and International Health, Graduate School of Medicine, University of the Ryukyus, Okinawa, Japan; 8 Department of Global Health, School of Health Sciences, University of the Ryukyus, Okinawa, Japan; 9 Bureau of International Cooperation, National Center for Global Health and Medicine, Tokyo, Japan; 10 Department of Nursing, Faculty of Health and Welfare, Seinan Jo Gakuin University, Fukuoka, Japan

**Keywords:** Community health worker, Social capital, Performance, Lao PDR

## Abstract

**Background:**

Improving the performance of community health workers (CHWs) is a global issue. The relationship between CHWs and their community may impact their performance. In Lao People’s Democratic Republic (Lao PDR), CHW are called village health volunteers (VHV). Lao PDR has a problem with VHV inactivity, especially in rural areas. This study focused on which aspects of social capital are related to VHV performance.

**Methods:**

This research represents a cross-sectional study with a quantitative survey based primarily on interviews using a semi-structured questionnaire. Interviews were conducted with 149 VHVs living and working in the Sepon District. VHV performance evaluation was measured with scores on a 5-point scale, and the cutoff point for designating performance as good or poor was set at the median score. This evaluation of VHV performance was conducted as a self-evaluation by VHVs and by health center staff who were supervisors of the VHVs. Measurement of social capital was accomplished using the short version of the Adapted Social Capital Assessment Tool (SASCAT). For statistical analyses, logistic regression was used to calculate adjusted odds ratios (OR) and 95% confidence intervals (CI).

**Results:**

The results of multiple logistic regression adjusted by moderator variables showed that citizenship activities in the structural social capital component of SASCAT were significantly related to performance in self-evaluation by VHVs (adjusted OR: 2.10, 95% CI: 1.19-3.71) and the evaluations by health center staff (adjusted OR: 1.67, 95% CI: 1.01-2.77). Support from groups (adjusted OR: 1.87, 95% CI: 1.27-2.76) and cognitive social capital (adjusted OR: 7.48, 95% CI: 2.14-26.10) were found to be significantly associated but only for VHV self-evaluation.

**Conclusions:**

The results suggest that individuals who interact with important figures in the community and who cooperate with other villagers whenever problems arise, i.e., have social capital, exhibit good performance as VHVs. These findings suggest that increasing citizenship activities could increase the retention rate of CHWs and help improve their performance. Citizenship activities could also be used as a predictive indicator when selecting new CHWs.

## Background

According to documents issued by the WHO [1], the term *community health worker* (CHW) refers to local healthcare workers who are not medical professionals such as doctors or nurses but who provide some form of healthcare service to the public after receiving required training. Although the titles and tasks performed by CHWs differ from country to country, the role that such workers play in local healthcare systems is universal [1]. At present, as efforts to achieve the Millennium Development Goals (MDGs), scheduled to end in 2015, reach a climax, it has been pointed out that improving the robustness of existing healthcare systems [2] and enhancing basic healthcare services are important to achieving goals related to health indicators [3,4]. From that standpoint, it has been demonstrated that policies utilizing CHWs are effective in improving health indicators and that such strategies also excel in terms of cost-effectiveness [1,5,6].

At the same time that attention is being paid to the role of CHWs, certain challenges have emerged regarding their performance. A review of the literature concluded that CHWs did not consistently provide services that are likely to have substantial effects on health and that quality was usually poor [7]. Furthermore, such ineffective performance has variously been attributed to the lack of monetary incentives, appropriate supervision, or ongoing training [8-10].

The Lao People’s Democratic Republic (hereafter, “Lao PDR”) is a landlocked country that is considered the least developed country in Asia. Recently, the majority of health indicators in this country have improved but some of these have still not reached the targets of the MDGs. For example, infant mortality rate is 68 (per 1,000 live births) and prevalence of stunting children under-5 years of age is 44.2% [11]. According to the world health statistics provided by WHO [12], it is estimated that 44% of the population lives below the poverty line of $1/day, and approximately 63% of the country’s population is said to reside in non-urban areas. That can cause difficulty of access to basic health services and lead to a widening gap of health indicators between urban and rural areas [11]. In Lao PDR, CHWs are referred to as village health volunteers (VHVs). The government has been trying to improve the access to basic health services, especially for people who live in rural areas, and anticipated VHVs would perform a crucial role as local health service practitioners in the policy [13]. Several individuals in each village are selected to function as VHVs according to criteria such as being healthy, having a primary or higher level of education and being willing to take the position on a voluntary basis. As in the international community in general, problems exist related to the performance of VHVs in Lao PDR. The low submission rate of monthly reports has been identified as a problem in previous research [14]. The concern regarding CHW performance has started to garner attention in Lao PDR.

*Social capital* is a sociological term but is used in a wide array of disciplines, including economics and epidemiology. Certain aspects of the definition of social capital differ slightly from researcher to researcher. According to Putnam *et al*. [15], social capital refers to features such as trust, norms, and networks which can generate some social benefit. While the categorization of social capital also tends to differ among researchers, two of the most basic categories are *collective social capital,* referring to features of groups such as communities, and *individual social capital,* referring to the features of individuals, with each having its own methods of measurement [16]. Viewing social capital from another dimension, Krishna and Shrader divide it into *structural social capital,* referring to tangible organizations as well as their activities and networks, and *cognitive social capital,* referring to the intangible reciprocity between community members in the form of trust, sharing of social norms, etc. [17].

In previous research on the relationship between CHWs and social capital in Colombia, Robinson and Larsen [18] suggest that the relationship between CHWs and the community may impact CHW performance. Also, Kratzke *et al*. [19] identified social norms, which are a component of social capital, to be a factor contributing to the increased use of mammograms as part of efforts by CHWs to promote health. Furthermore, in an assessment of a CHW program in India, Gopalan *et al*. [20] reported that social responsibility impacts the performance motivation of CHWs. Based on this and other research, it can be surmised that a CHW’s performance is somehow related to the social capital in their possession. However, little attention has been given to the association between the social capital of CHWs and their performance.

We hypothesized that VHVs who have high social capital tend to exhibit high standards of performance in their activities. The objective of this study was to test the association of VHVs’ social capital with their performance by using a social capital assessment tool.

## Methods

### Study site and population

**Figure 1:**
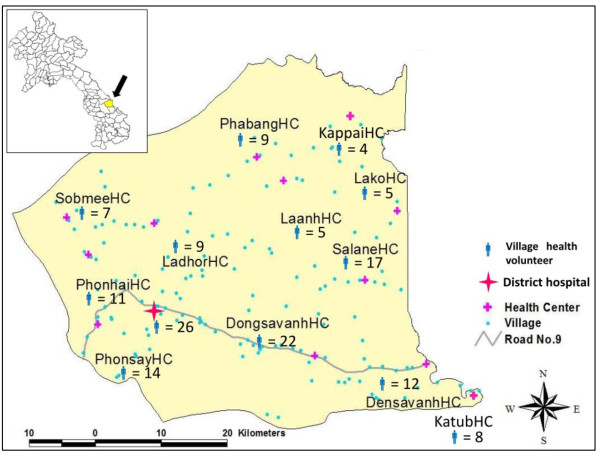
Map and position of district health office (DHO) and 12 health centers (HC) in the Sepon district.

The study was conducted in the Sepon District, Savannakhet Province, Lao PDR. The Sepon District is located about 500 km southeast of Vientiane, the capital, near the Vietnamese border. According to the Poverty Reduction Strategy Paper published by the Lao government, 47 of the 137 districts, including Sepon District, have been designated as impoverished based on various criteria such as income and education, healthcare services, access to safe water, etc. [21]. As of 2012, the population of the Sepon District was approximately 48,000, of which 75% represented ethnic minorities. In terms of public healthcare service providers, there is one district health department (which operates a district hospital) and 12 health centers in the district (Figure 1). These 13 agencies are responsible for managing the VHVs who are responsible for each catchment area.

The inclusion criterion for the target population was VHVs who are registered with the Sepon District Health Office. The exclusion criterion was VHVs who had less than six months’ experience. After applying these criteria, we chose 150 out of 161 VHVs in Sepon District, and we were able to conduct interviews with 149.

This research was conducted upon approval of the Ethics Review Board at the Nagasaki University Graduate School of International Health Development and subsequent acknowledgement thereof by the National Ethics Committee for Health Research in Lao PDR. We also explained the objectives of this study to all participants before the interview, and obtained their signature as consent.

### Study design and data collection

A quantitative, cross-sectional survey based primarily on interviews using a semi-structured questionnaire was conducted in September and October of 2012. To collect some data about VHVs’ social capital and socio-demographic and economic characteristics, trained interviewers conducted interviews with VHVs of each target village. VHV performance was collected based on two criteria: self-evaluation of performance by the VHVs and evaluation of performance by health center staff as their supervisors. Thirteen individuals were selected supervisors, one from each health center and one from the district health office, and they were asked to evaluate the performance of VHVs in their respective catchment areas.

According to the Savannakhet Provincial Health Department, VHVs in each village are expected to engage in the following tasks related to resident healthcare:

1) assisting health center staff members in outreach activities

2) imparting health education to villagers

3) providing basic treatment

4) facilitating prenatal care

5) malarial surveillance and case management

6) vital event surveillance

We excluded activities 3) and 5) from the analysis because these activities are not conducted by all VHVs. Activity 6) was also excluded because more than 90% of VHVs regularly reported vital event information, and we determined that this activity was successfully conducted by VHVs both with and without social capital.

### Performance evaluation

In previous research led by Stekelenburg *et al.*[22] and Kalyango *et al*. [23], records or reports about CHW activities were collected to assess performance. However, according to the authority of the Sepon District Health Office, VHVs have not been called on to submit or record their activities so far. Therefore, we followed the approach of Kawakatsu *et al*. [24] and Alam *et al*. [25], and interviewed health center staff who act as VHV supervisors to evaluate VHV performance. Also, we collected VHV self-evaluations because health center staff may not be familiar with the role of VHVs as they do not regularly review VHV activity.

The self-evaluations and evaluations of VHV performance by health center staff entailed the assignment of scores on a 5-point scale (5 = very active; 4 = active; 3 = neither active nor inactive; 2 = inactive; 1 = never worked) with regard to the three VHV task areas. The cutoff point for designating performance as good or poor was set at the median score. A binary outcome variable was created by designating the upper 50% of scores as good performance and the lower 50% of scores as poor performance. Cronbach’s alpha was calculated to evaluate the reliability of measures used in the pretest. For self-evaluations by VHVs, the result was 0.70 and for the performance evaluation by health center staff it was 0.78.

### Social capital

Social capital was measured using the short version of the Adapted Social Capital Assessment Tool (SASCAT). The SASCAT was developed based on the Adapted Social Capital Assessment Tool (ASCAT) proposed by Harpham *et al*. [26] (Table 1). In addition, the construct validation of the SASCAT has been demonstrated in Vietnam and Peru [27,28]. The SASCAT is a questionnaire comprising nine questions in five domains that focus on two components of social capital—structural social capital and cognitive social capital. Each question is posed as a yes/no question, with “yes” answers receiving a score of 1 and “no” answers receiving a score of 0.

**Table 1:** Short version of the Adapted Social Capital Assessment Tool (SASCAT)

**Questions**	**Coding/score range**
***Group membership***	
1. In the last 12 months, have you been an active member of any of the following type of groups in your village?	Score between 0 and 7
• Mass organization	
• Farmer/Agriculture/Livestock association	
• Political group	
• Religious group	
• Credit/funeral group	
• Sports group	
• Other	
***Support from groups***	
2. In the last 12 months, did you receive from the group any emotional help, economic help or assistance in helping you know or do things?	Score between 0 and 7
• Mass organization	
• Farmer/Agriculture/Livestock association	
• Political group	
• Religious group	
• Credit/funeral group	
• Sports group	
• Other	
***Support from individual***	
3. In the last 12 months, did you receive any support or help from any one of the following, this can be emotional help, economic help or assistance in helping you know or do things?	Score between 0 and 9
• Family	
• Neighbors	
• Friends who are not neighbors	
• Village leaders	
• Religious leaders	
• Politicians	
• Government officials/civil service	
• Charitable organizations/NGOs	
• Other	
***Citizenship activities***	
4. In the last 12 months, have you joined together with other village members to address a problem or common issues?	Yes = 1, No = 0
5. In the last 12 months, have you talked with local authority or governmental organization about problems in this village?	Yes = 1, No = 0
***Cognitive social capital***	
6. In general, can the majority of people in this community be trusted?	Yes = 1, No = 0
7. Do the majority of people in this village generally get along with each another?	Yes = 1, No = 0
8. Do you feel as though you are really a part of the village?	Yes = 1, No = 0
9. Do you think that a majority of the people in the village would try to take advantage of you if they got the chance?	Yes = 0, No = 1

### Statistical analysis

Statistical analyses were performed using IBM SPSS statistics 20. Logistic regression was used in bivariate and multivariate analyses to calculate odds ratios (OR) (adjusted OR in multivariate analysis) and 95% confidence intervals (95% CI). Variance inflation factors (VIF) were calculated to detect the presence of multicollinearity. In this study, when VIF was less than 10.0 as a familiar cutoff value, it indicated that there was no multicollinearity.

## Results

### Study population attributes

Of the 149 VHVs, the mean age was 38.4 years (range, 16-58 years) (Table 2). The study group included 133 males (89.3%). One hundred and forty-three individuals (96.0%) were engaged in agriculture, and of the 144, (96.6%) individuals meet the educational requirement of being a VHV which is having a primary or higher level of education. Of the VHVs interviewed, 108 (72.5%) possessed a motorcycle, while 35 (23.5%) did not own any motorized vehicle. The mean number of years (±SD) of experience as a VHV was 7.8 years (±4.7). The mean distance from villages to the nearest health center was 8.1 km (±6.8). The mean number of training sessions attended in a year was 2.95 (±2.0).

**Table 2:** Characteristics of 149 village health volunteers in the Sepon district

**Characteristics**	**VHVs (n = 149)**	**%**
Sex		
Male	133	89.3
Female	16	10.7
Age		
<30	47	31.5
30 ~ 44	59	30.6
>44	43	28.9
Main job		
Famer	143	96.0
Merchant	3	2.0
Other	3	2.0
Education attainment		
No formal education	5	3.4
Primary (1-5 years)	114	76.5
Secondary or above	30	20.1
Possession of vehicles*		
Nothing	35	23.5
Motorbike	108	72.5
Car or tractor	48	32.2
Distance between village and health center (Km)		
<5	55	36.9
5 ~ 9	49	32.9
>9	45	30.2
Work experience as VHV (years)		
<5	38	25.5
5 ~ 9	60	40.3
>9	51	34.2
Number of training sessions attended		
<2	37	24.8
2 or 3	66	44.3
>3	46	30.9

*Multiple responses.

### Social capital

With regard to scores for each item related to individual social capital, the maximum observed score for group membership was 6 out of a possible 7. Similarly, the maximum observed score for support from individuals was 8 out of a possible 9. Mean scores for group membership, support from groups, and support from individuals were 2.8 (±1.3), 2.3 (±1.5), and 4.7 (±1.8), respectively. The mean score for citizenship activities was 1.3 (±0.8). On questions related to cognitive social capital, 117 (78.5%) individuals scored 4 out of 4, with a mean score of 3.7 (±0.7).

### Performance evaluations

While the theoretical minimum and maximum total scores for VHV performance evaluations are 4 and 20, the scores observed for self-evaluations by VHVs were 9 and 20, with a mean score of 15.7 (±2.40). In the performance evaluations by health center staff, the minimum and maximum scores observed were 4 and 20, with a mean score of 13.9 (±3.54). The tendency of VHVs to assign themselves higher performance scores than given to them by the health center staff can also be seen in this analysis of total performance scores. Cutoff values to differentiate “good” and “poor” performance were set as the median scores for each evaluating group. Cutoff values were 16 in the case of the VHV self-evaluations and 14 in the case of evaluations by health center staff. As a result, in the case of VHV self-evaluations, 65 individuals were designated as good performers, whereas 84 were identified as poor performers. The same figures for health center staff evaluations were 60 and 89, respectively.

### Association between social capital and performance

The results of bivariate logistic regression of self-evaluations and evaluations by health center staff of VHV performance against individual variables are presented in Table 3. In the case of self-evaluations by VHVs, educational attainment (OR: 2.28, 95% CI: 1.08-4.80), number of training sessions attended (OR: 1.75, 95% CI: 1.11-2.76), support from groups (OR: 1.37, 95% CI: 1.08-1.73), citizenship activities (OR: 2.40, 95% CI: 1.51-3.80), and cognitive social capital (OR: 6.11, 95% CI: 2.08-18.00) were found to be significantly related to performance. In the case of evaluations by health center staff, number of training sessions attended (OR: 1.71, 95% CI: 1.08-2.71), support from groups (OR: 1.25, 95% CI: 1.00-1.58), and citizenship activities (OR: 1.70, 95% CI: 1.10-2.61) were found to be significantly related to performance.

**Table 3:** Bivariate logistic regression for associations between variables and the evaluation of village health volunteers’ performance

**Variables**	**Village health volunteers**		**Health center staff**	
	**Odd ratio**	**95% Confidence interval**	**Odd ratio**	**95% Confidence interval**
Age	1.30	0.85-1.98	1.18	078-1.80
Distance between village and health center	1.20	0.80-1.78	1.00	0.67-1.50
Education attainment	2.28	1.08-4.80	1.96	0.94-4.08
Number of training sessions attended	1.75	1.11-2.76	1.71	1.08-2.71
Structural social capital				
Group membership	1.17	0.91-1.50	1.19	0.92-1.54
Support from groups	1.37	1.08-1.73	1.25	1.00-1.58
Support from individuals	1.13	0.94-1.35	1.10	0.91-1.32
Citizenship activities	2.40	1.51-3.80	1.70	1.10-2.61
Cognitive social capital				
Cognitive social capital	6.11	2.08-18.00	1.64	0.90-2.80

The results of multiple logistic regression of VHV performance against social capital variables are presented in Table 4. In the case of self-evaluations by VHVs, support from groups (adjusted OR: 1.87, 95% CI: 1.27-2.76), citizenship activities (adjusted OR: 2.10, 95% CI: 1.19-3.71), and cognitive social capital (adjusted OR: 7.48, 95% CI: 2.14-26.10) were found to be significantly related to performance. In the case of evaluations by health center staff, only citizenship activities (adjusted OR: 1.67, 95% CI: 1.01-2.77) were found to be significantly related to performance.

**Table 4:** Multiple logistic regression for associations between social capital and the evaluation of village health volunteers’ performance

**Variables**	**Village health volunteers**		**Health center staff**	
	Adjusted odd ratio*	**95% Confidence interval**	Adjusted odd ratio*	**95% Confidence interval**
**Structural social capital**				
Group membership	0.89	0.60-1.32	1.05	0.73-1.51
Support from groups	1.87	1.27-2.76	1.29	1.92-1.80
Support from individuals	0.78	0.59-1.04	0.84	0.66-1.08
Citizenship activities	2.10	1.19-3.71	1.67	1.01-2.77
**Cognitive social capital**				
Cognitive social capital	7.48	2.14-26.10	1.28	0.65-2.52

*Adjusted by age, distance between village and health center, education attainment, number of training sessions attended.

## Discussion

### Association of individual social capital and village health volunteer activities

Although self-evaluations by VHVs and evaluations by health center staff yielded different results, one variable was observed to be significantly related to performance in both cases, namely engagement in “citizenship activities,” which is a component of structural social capital. The “citizenship activities” item includes two questions regarding “joining together with other village members” and “talking with a local authority or government organization.” In other words, it appears that individuals who have connections with important figures in the community and who cooperate with other villagers whenever problems arise exhibit good performance as VHVs.

According to previous studies [22,29], community support for CHW activity is a very important factor for improving CHW performance. In order to assist the health center staff and conduct health education, VHVs need the cooperation and consent of all villagers. Therefore, it is likely that VHVs who interact with other villagers might get cooperation from villagers more easily. Furthermore, VHVs who interact with important figures in the community might have a sense of social responsibility. Kane *et al*. [30] reported that perception of improvement in social status and having a valuable social role can be factors contributing to improved VHV performance. However, performance and social capital are interactive because VHV activities are a public role which can be interpreted as citizenship activities. It was considered that VHV performance also had effect on citizenship activities. In the case of self-evaluation by the VHVs, “support from groups” and “cognitive social capital” were found to be related to performance. The question about “support from groups” asks whether the VHV receives support from various formal groups. In brief, the result that “support from groups” was associated significantly reinforces the finding that VHVs who get cooperation from village members or groups might improve their performance. As for cognitive social capital, the study led in India [20] reports that CHWs’ social responsibility might have a positive effect on improving performance motivation. Because trust and reciprocity are prominent features of cognitive social capital, the presence of these factors tends to promote compliance with the roles entrusted to VHVs. It can be reasonably argued that these factors have an impact on performance, as shown by Gopalan *et al*. [20]. Furthermore, according to a review of the literature by Eriksson [31], formal networks tend to encourage reciprocity. From this, we believe that VHVs who receive support from various groups also have high cognitive social capital in a sense of reciprocity and trust with villagers.

### Factors other than social capital

Since the study was conducted in Sepon district only, we assumed that the effects of other parts of contributors such as the supervisory system and the structure of the financial incentive [9] were similar among the VHVs who participated in our study. However, another variable, “number of training sessions attended”, was also significantly associated with VHV performance in the bivariate logistic regression analysis. Although it may infer reverse causation, other study has also reported that the adequate training programs for CHWs improve their performance [29]. Therefore, we are aware that other factors might have affected on VHV performance as well as social capital. Also, we believe that social capital and other non-monetary incentives deserve more attention in terms of the sustainability and feasibility of the program.

### Limitations

In this study, we have several limitations. The main limitation of this study is that performance evaluation measures could not be validated in the study. However, the items of the measures were considered to be appropriate because these were established based on VHV official job descriptions, and they were developed by the researchers who conducted investigations about VHVs and by local authorities in the Sepon District Health Office and Savannakhet Provincial Health Department. As a strategy for moving forward, we plan to re-interview a small subsample of VHVs (20 or less) to determine the degree to which self-evaluations of performance by the same individual vary. By assessing test-retest reliability, it will be possible to confirm measurement precision.

Secondly, as the self-evaluation of performance by VHVs is potentially highly biased, caution is necessary when interpreting the results of VHV self-evaluations. Despite the limitation, the self-evaluation can contribute to complementing the evaluations by health center staff, as they might have difficulty in realizing some VHV activities, e.g., informal health education activities through chatting with villagers.

The third limitation is the generalizability of the sample. This study was conducted in the Sepon District only. We need to expand the research to other districts in order to include more subjects. Also, it might be difficult to apply the same results to the whole country because people living in the Sepon District are mainly ethnic minorities.

The final limitation is related to the statistical methods. We could not use multiple linear regression which would have been more appropriate to the analyses because our data did not meet the assumptions to enable us to use the analyses. Therefore, we used medians as the cut-off points in order to define poor or good performance. In doing so, results might have changed if other cut-off values were applied. However, given our sample size and data, we believe that it was reasonable method to use the median score in this pilot study setting. Also, we focused only on individual social capital in this study though it is considered that collective social capital is also related to VHV performance. Multi-level analysis is thus needed in further study.

Due to having these limitations, the study result lacks robustness despite careful design and execution. Hence, additional investigation is needed to further confirm results by developing the design, including evaluation by beneficiaries of VHV activities such as villagers, and expanding the study site and population.

### Recommendations

In summary, we recommend two methods for improving VHV performance based on the strengthening of structural (citizenship activities and support from groups) and cognitive social capital. The first recommendation is to scrutinize the structural and cognitive social capital of candidates when selecting VHVs. In a study of CHWs in Zambia [22], researchers found that lack of cooperation by residents toward CHW activities resulted in reduced CHW performance and emphasized the importance of selecting appropriate CHWs capable of cooperating with residents. Current criteria for choosing new VHVs adopted by the Lao government is considered to be working well. However, we believe that it will be useful to examine candidates’ track records of cooperation with residents, relationships with important community members or governors, and recognition of the importance of mutual aid as criteria for selection of VHVs.

The second recommendation is to strengthen the structural and cognitive social capital of individuals currently functioning as VHVs. Kane *et al*. [30] concluded that strengthening CHWs’ roles in communities can lead to improved performance, citing as an example the case of an intervention involving the community as a whole in which the CHW came to function as a role model in the community. According to Anirudh and Norman [32], cognitive social capital and structural social capital are interrelated, with the former being reproduced by the latter. In other words, the strengthening of structural social capital can be expected to increase the cognitive social capital of individuals already engaged as VHVs.

## Conclusions

We can conclude that the results of this research are useful, if only for the reason that they represent a first attempt to study the relationship between social capital and VHV performance in Lao PDR and have led to the proposal of effective methods to improve VHV performance. Furthermore, in the greater context of global health, few previous studies on the relationship between social capital and CHW performance have yielded effective policy recommendations.

The findings of this study suggest the possibility that social capital could be used for improving CHW performance. First, social capital could work as a predictive indicator to help find VHVs who will perform well. It may be useful when selecting a new VHV from a village. Another point is that VHV performance could be improved by fostering their social capital. It will prove useful not only in Lao PDR but also in other developing countries where health policy incorporating CHWs is being implemented. This is because CHWs play an important role in enhancing the community health of low- and middle-income countries and because improvement of CHW performance is an ongoing policy challenge in these countries. Finally, we believe that continued efforts to deepen our understanding of the association between social capital and CHWs will prove valuable in the future.

## Competing interests

The authors declare that they have no competing interests.

## Authors’ contributions

YS was substantially involved in the conception and design of this study as well as data analysis and data collection. YS was also involved in writing and revising the manuscript and has approved this final version. TP was involved in the conception and the design of this study as a local authority. DN was involved in writing and revising the manuscript, especially to perform statistical analysis. SK was involved in arranging the schedule to submit the proposal to the ethical committee in Lao PDR and to read and comment on the draft manuscript. PP and PN were involved in the coordination of the research schedule as local authorities at the provincial and district levels. KM was involved in the coordination and the negotiation with local authorities during this study. YK, MS and JK were substantially involved in data analysis and writing this manuscript as supervisors, and JK was also involved in the whole design of this study as the main supervisor. All authors read and approved the final manuscript.

## Pre-publication history

The pre-publication history for this paper can be accessed here:

http://www.biomedcentral.com/1472-6963/14/123/prepub
